# Topically Applied Taurine Chloramine Protects against UVB-Induced Oxidative Stress and Inflammation in Mouse Skin

**DOI:** 10.3390/antiox10060867

**Published:** 2021-05-28

**Authors:** Seong Hoon Kim, Hye-Won Yum, Seung Hyeon Kim, Su-Jung Kim, Kyeojin Kim, Chaekyun Kim, Young-Ger Suh, Young-Joon Surh

**Affiliations:** 1 Research Institute of Pharmaceutical Sciences, College of Pharmacy, Seoul National University, Seoul 08826, Korea; daduhoon@snu.ac.kr (S.H.K.); uknowyum@hanmail.net (H.-W.Y.); shjbeh@naver.com (S.H.K.); nynna79@snu.ac.kr (S.-J.K.); kyeojin01@gmail.com (K.K.); 2 Cancer Research Institute, Seoul National University, Seoul 03087, Korea; 3 Department of Pharmacology and Toxicology, College of Medicine, Inha University, Incheon 22212, Korea; chaekyun@inha.ac.kr; 4 College of Pharmacy and Institute of Pharmaceutical Sciences, CHA University, Seongnam 13488, Korea; ygsuh@snu.ac.kr; 5 Department of Molecular Medicine and Biopharmaceutical Sciences, Graduate School of Convergence Science and Technology, Seoul National University, Seoul 08826, Korea

**Keywords:** taurine, taurine chloramine, skin inflammation, heme oxygenase-1, STAT3, Nrf2

## Abstract

Excessive exposure to solar light, especially its UV component, is a principal cause of photoaging, dermatitis, and photocarcinogenesis. In searching for candidate substances that can effectively protect the skin from photodamage, the present study was conducted with taurine chloramine (TauCl), formed from taurine in phagocytes recruited to inflamed tissue. Irradiation with ultraviolet B (UVB) of 180 mJ/cm^2^ intensity caused oxidative damage and apoptotic cell death in the murine epidermis. These events were blunted by topically applied TauCl, as evidenced by the lower level of 4-hydroxynonenal-modified protein, reduced proportions of TUNEL-positive epidermal cells, and suppression of caspase-3 cleavage. In addition, the expression of two prototypic inflammatory enzymes, cyclooxygenase-2 and inducible nitric oxide synthase, and transcription of some pro-inflammatory cytokines (*Tnf, Il6, Il1b, Il10*) were significantly lower in TauCl-treated mice than vehicle-treated control mice. The anti-inflammatory effect of TauCl was associated with inhibition of STAT3 activation and induction of antioxidant enzymes, such as heme oxygenase-1 and NAD(P)H:quinone oxidoreductase 1, through activation of nuclear factor erythroid 2-related factor 2.

## 1. Introduction

The skin is the largest organ constituting the outermost part of the human body, and it is vulnerable to external stressors [1,2,3]. Despite the important role of skin, intractable skin diseases are gradually increasing in modern society. Solar radiation is associated with the majority of skin disorders. Acute exposure to sunlight accounts for erythema/sunburn, tanning, and allergic contact dermatitis, whereas chronic exposure causes actinic keratosis, immunosuppression, premature aging of the skin, and melanoma [4,5]. Among the ultraviolet rays, ultraviolet A (UVA) and ultraviolet B (UVB) are the main causes of oxidative stress and inflammation on the skin [6]. UVA has weak energy intensity, but when exposed for a long period of time, it can provoke skin erythema, wrinkles, and pigmentation. UVB directly damages the skin, causing apoptosis, inflammation, and photoaging [7,8], and can also induce photocarcinogenesis [9,10]. Adequate exposure to sunlight helps to synthesize vitamin D, which facilitates calcium metabolism, improves immunity, supports cellular redox balance, and maintains homeostasis [8,11,12]. However, unbalanced and excessive production of reactive oxygen species (ROS), as a consequence of severe exposure to solar radiation, triggers various types of inflammatory responses.

Human bodies are equipped with a distinct set of substances that counteract oxidative stress and inflammatory tissue damage. Taurine, an amino sulfonic acid, is one such compound that has both antioxidant and anti-inflammatory properties (reviewed in [13] and referenced therein). It also plays a vital role in various physiological processes [14]. Taurine is ubiquitously present in mammalian cells and tissues, and it is present at high concentrations in organs, such as the liver, heart, kidneys and brain, as well as in skeletal muscle and blood cells. Since the activity of cysteine sulfinate decarboxylase involved in the biosynthesis of taurine is low in humans, taurine is mainly supplied as part of a food or dietary supplement. It is abundant in fish and seafood and can also be obtained from meat and dairy products [15].

Human leukocytes contain 20–50 mM of taurine, and when inflammation occurs, its concentration in the inflamed site and surrounding tissues further increases [16]. Activated phagocytes, such as neutrophils, produce hydrogen peroxide (H_2_O_2_) in the process of defending against invading pathogens, which, in the presence of chloride, produces hypochlorous acid (HOCl) by myeloperoxidase (MPO) activity. HOCl then reacts with taurine to generate taurine chloramine (TauCl) that has strong anti-inflammatory as well as antioxidant properties [17]. The protective effects of TauCl on oxidative stress, metabolic syndrome, and inflammation have been reported [18,19]. In this study, we investigated the effect of topically applied TauCl on UVB-induced inflammation as well as oxidative stress in mouse skin.

## 2. Materials and Methods

### 2.1. Materials

The crystalline sodium salt form of TauCl (MW 181.57) was prepared as described previously [20]. The primary antibody for detecting protein modified with 4-hydroxynonenal (4-HNE) was purchased from the Japan Institute for the Control of Aging (JaICA), Nikken SEIL Co., Ltd. (Shizuoka, Japan). Primary antibodies for Signal transducer and activator of transcription (STAT3), P-STAT3^Y705^, cyclin D1, and lamin B1 were provided by Cell Signaling Technology (Danvers, MA, USA). Antibodies for detecting cyclooxygenase-2 (COX-2) and Nrf2 were purchased from Abcam (Cambridge, MA, USA). Antibodies for heme oxygenase-1 (HO-1) and inducible nitric oxide synthase (iNOS) were supplied by Enzo Life Sciences (Farmingdale, NY, USA) and BD Biosciences (San Jose, CA, USA), respectively. Antibodies for caspase-3 and its cleaved form were purchased from Cell Signaling Technology (Danvers, MA, USA).

### 2.2. Animals

Female SKH1-*Hr^hr^* mice (5 to 6 weeks of age) were purchased from Orient Bio Inc. (Seongnam-si, Korea). Mice were randomly divided into four groups (*n* = 3 per group). Female SKH1 hairless mice have been shown to be more sensitive to inflammatory response, as determined by skin fold thickness and myeloperoxidase activity, as compared with males [21]. Mice were housed in plastic cages under controlled conditions of temperature (23 ± 2 °C), humidity (50 ± 10%), and light (12/12 h light/dark cycle). All animal experiments complied with the Guide for the Care and Use of Laboratory Animals and were approved by the Institutional Animal Care and Use Committee (IACUC) at Seoul National University (IACUC number; SNU-160720-11-2).

### 2.3. UVB Irradiation and Treatment

For UVB irradiation, tubes (5 × 8 Watts) that emit wavelengths mainly in the UVB region, with a peak at 312 nm were used. The intensity of UVB irradiation to mouse skin was 180 mJ/cm^2^ as measured by a Biolink BLX-312 (Vilber Lourmat; Marne-la-Vallée, Paris, France). The hairless mice were topically administered TauCl (10 μmol) dissolved in 200 μL of water: propylene glycol: ethanol solution (2:1:2, *v/v*) or vehicle to the dorsal skin 30 min prior to UVB irradiation. The dorsal epidermis samples of the euthanized mice were collected 6 h later. To obtain dorsal epidermis, skin was grasped with forceps, and an incision was made with scissors from the tail, past the flank, to the neck. The tissues were stored at −70 °C until use.

### 2.4. Histology

Parts of collected skin were fixed with 10% phosphate-buffered formalin and embedded in paraffin. Each section was stained with hematoxylin and eosin (H&E). The H&E stained sections were examined under a light microscope (Nikon; Tokyo, Japan) to detect the presence of lesions.

### 2.5. Immunohistochemical Analysis

The dissected skin tissues were prepared for immunohistochemical analysis with antibodies directed against 4-HNE (JaICA; Shizuoka, Japan), COX-2 (Cell Signaling Technology; Danvers, MA, USA), and P-STAT3 (Cell Signaling Technology; Danvers, MA, USA). Five-micrometer sections of 10% formalin-fixed, paraffin-embedded tissues were deparaffinized and rehydrated in a series of xylene and ethanol. The deparaffinized sections were heated in a microwave and boiled twice for 6 min in a 10 mM citrate buffer (pH 6.0) for antigen retrieval. To diminish nonspecific staining, each section was treated with 3% hydrogen peroxide and 4% peptone casein blocking solution for 15 min. The slides were incubated with a diluted primary antibody at room temperature for 40 min in Tris-HCl-buffered saline containing 0.05% Tween 20, then incubated with a horseradish peroxidase-conjugated secondary antibody (Dako, Glostrup, Denmark). The tissues were treated with 3,3′-diaminobenzidine tetrahydrochloride to detect the peroxidase binding sites. Finally, counterstaining was performed using Mayer’s hematoxylin.

### 2.6. Terminal Deoxynucleotidyl Transferase dUTP Nick End Labeling (TUNEL) Assay

Apoptotic DNA fragmentation was detected by the TUNEL assay with the ApopTag^®^ Peroxidase In Situ Apoptosis Detection Kit (Chemicon; Temecula, CA, USA). The isolated skin tissues were rinsed with phosphate-buffered saline (PBS) and fixed in 10% buffered formalin (Sigma-Aldrich; Saint Louis, MO, USA) for the TUNEL assay. The apoptotic cells were visualized under a light microscope (Nikon; Tokyo, Japan).

### 2.7. Immunofluorescence Staining

The skin specimens were fixed, paraffin-embedded, and sectioned, and the sections were deparaffinized and rehydrated by serial washes with graded xylene and alcohol. For immunofluorescence staining, tissue sections were boiled in 10 mM of sodium citrate (pH 6.0) for antigen retrieval, subjected to serial washing, and permeabilized for 45 min at room temperature using 0.2% Triton X-100 in PBS and blocked with 3% bovine serum albumin (BSA) in PBS for 1 h at room temperature. The tissue sections were stained with primary antibodies for P-STAT3 and Nrf2 (Cell Signaling Technology; Danvers, MA, USA) and diluted at 1:250 in 3% BSA overnight at 4 °C. After washing three times with PBS each 5 min to remove primary antibodies, tissues were incubated with appropriate secondary antibodies. Nuclei were counterstained with DAPI (Invitrogen; Carlsbad, CA, USA). Immunofluorescence images were collected on a fluorescence microscope (Nikon; Tokyo, Japan).

### 2.8. Tissue Lysis and Protein Extraction

The UVB-irradiated dorsal skin of mice was collected as described above, and fat was removed on ice to attain the epidermis. Collected epidermis was homogenized with the lysis buffer (20 mM Tris-HCl (pH 7.5), 150 mM NaCl, 1 mM Na_2_EDTA, 1 mM EGTA, 1% Triton, 2.5 mM sodium pyrophosphate, 1 mM β-glycerophosphate, 1 mM Na_3_VO_4_, 1 μg/mL leupeptin, 1 mM phenylmethyl sulfonylfluoride (PMSF), and an EDTA-free cocktail tablet) in an ice bath. The lysates were vortexed every 10 min for 3 h on the ice, followed by centrifugation (13,000× *g*, 15 min at 4 °C). The supernatants were collected and stored at −70 °C until use.

### 2.9. Preparation of Cytosolic and Nuclear Extracts

The fat-free dorsal skin tissues were homogenized with 1 mL buffer A (10 mM 4-(2-hydroxyethyl)-1-piperazineethanesulfonic acid (HEPES, pH 7.8), 1.5 mM MgCl_2_, 10 mM KCl, 0.5 mM dithiothreitol (DTT), 0.2 mM PMSF) followed by vortex-mixing every 10 min for 3 h in an ice bath. The lysates were mixed with 0.1% Nonidet P-40 (NP-40) 30 min before centrifugation. After centrifugation at 13,000× *g* for 15 min, the supernatants (the cytosolic extracts) were collected. The precipitated nuclear pellets were washed three times with buffer A containing 0.625% NP-40 to remove a residual cytosolic fraction. Nuclear pellets were then resuspended in 300 μL of buffer C (20 mM HEPES (pH 7.8), 420 mM NaCl, 1.5 mM MgCl_2_, 0.2 mM EDTA, 0.5 mM DTT, 0.2 mM PMSF and 20% glycerol). The nuclear lysates were vortexed every 10 min for 1 h, followed by centrifugation at 13,000× *g* for 15 min. The supernatants (nuclear extracts) were collected and kept at −70 °C until use.

### 2.10. Western Blot Analysis

The protein concentration in lysates was measured by using the Pierce^TM^ BCA Protein Assay Kit (Thermo Fisher Scientific; Rockford, IL, USA). The protein lysates were loaded on 7–15% SDS-polyacrylamide gel, and electrophoresis was performed under reducing conditions. Protein was transferred to the polyvinylidene difluoride membrane (PALL Life Sciences; Washington, NY, USA). The membranes were blocked with 5% non-fat, dry milk in Tris-buffered saline containing 0.1% Tween 20 (TBST) for 1 h at room temperature. After overnight incubation at 4 °C with diluted primary antibodies, the membranes were washed three times with TBST buffer for 10 min each. Then, membranes were incubated with a 1:5000 dilution of horseradish peroxidase-conjugated secondary antibody (Invitrogen; Carlsbad, CA, USA) for 1 h at room temperature, and washed again three times with TBST buffer. The protein expression was visualized with an enhanced chemiluminescent (ECL) detection kit (Absignal) (Abclon; Seoul, Korea) and LAS-4000 image reader (Fujifilm; Tokyo, Japan).

### 2.11. Real-Time Quantitative PCR (qPCR)

Total RNA was isolated from mouse skin tissues using TRIzol^®^ reagent (Invitrogen; Carlsbad, CA, USA), according to the manufacturer’s protocol. To synthesize the complementary DNA (cDNA), 1 μg of total RNA was reverse-transcribed with Moloney murine leukemia virus reverse transcriptase (Promega; Madison, WI, USA) for 50 min at 42 °C and again for 15 min at 72 °C. qPCR analysis was carried out in a 7500 Fast Real-Time PCR Instrument System (Applied Biosystems; Foster City, CA, USA) using the RealHelix^TM^ qPCR Kit (Green) (NanoHelix Co., Ltd.; Daejeon, Korea). The primers used for each qPCR reactions are as follows: *Il6*, 5′-TCT ATA CCA CTT CAC AAG TCG GA-3′ and 5′-GAA TTG CCA TTG CAC AAC TCT TT-3′; *Ptgs2*, 5′-TTC CAA TCC ATG TCA AAA CCG T-3′ and 5′-AGT CCG GGT ACA GTC ACA CTT-3′; *Nos2*, 5′-GTT CTC AGC CCA ACA ATA CAA GA-3′ and 5′-GTG GAC GGG TCG ATG TCA C-3′; *Tnf*, 5′-CAG GCG GTG CCT ATG TCT C-3′ and 5′-CGA TCA CCC CGA AGT TCA GTA G-3′; *Il1b*, 5′-TTC AGG CAG GCA GTA TCA CTC-3′ and 5′-GAA GGT CCA CGG GAA AGA CAC-3′; *Il10*, 5′-GCT GGA CAA CAT ACT GCT AAC C-3′ and 5′-ATT TCC GAT AAG GCT TGG CAA-3′; *Hmox1*, 5′-AGG TAC ACA TCC AAG CCG AGA-3′ and 5′-CAT CAC CAG CTT AAA GCC TTC T-3′; *Nqo1*, 5′-AGG ATG GGA GGT ACT CGA ATC-3′ and 5′-TGC TAG AGA TGA CTC GGA AGG-3′; *Gclc*, 5′- GGC TAC TTC TGT ACT AGG AGA GC-3′ and 5′- TGC CGG ATG TTT CTT GTT AGA G-3′; *Gss*, 5′- CCC ATT CAC GCT TTT CCC CT-3′ and 5′-GGG CAG TAT AGT CGT CCT TTT TG-3′; *Gapdh*, 5′-AGG TCG GTG TGA ACG GAT TTG-3′ and 5′-TGT AGA CCA TGT AGT TGA GGT CA-3′ (forward and reverse, respectively). The analysis was performed in a 20 μL final volume of reaction mixtures containing 10 μL of 2X Premix (Green) and 300 nM of each primer (forward and reverse). After an enzyme activation at 95 °C for 15 min, 40 cycles for amplification were performed at 95 °C for 20 s for denaturation and at 60 °C for 40 s for annealing, extension, and fluorescence acquisition.

### 2.12. Statistical Analysis

All the values were expressed as the mean ± standard deviation (SD) of at least three independent experiments. Statistical significance was determined by the Student’s *t*-test, or one-way ANOVA, followed by Tukey’s multiple comparisons for post-hoc test, and *p* < 0.05 was considered to be statistically significant. All the statistical analyses were applied using GraphPad Prism 8.0 (GraphPad Software; San Diego, CA, USA).

## 3. Results

### 3.1. UVB-Induced Oxidative Stress and Apoptosis Were Attenuated in TauCl-Treated Mice

**Figure 1:**
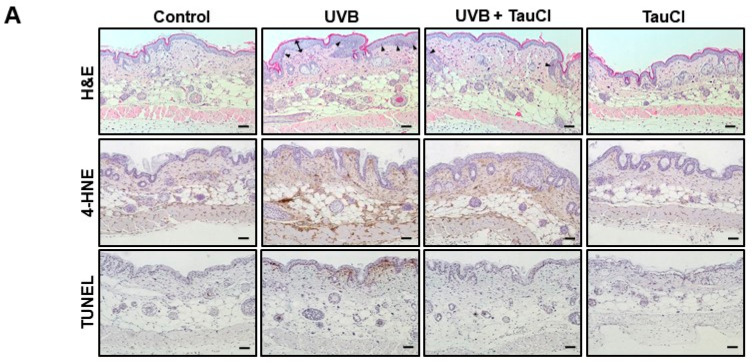

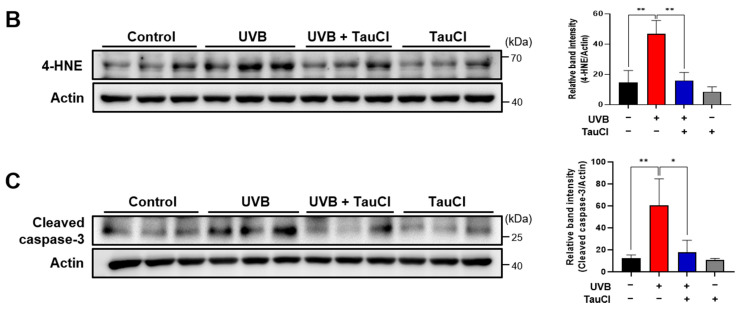
UVB-induced oxidative stress and apoptotic cell death were ameliorated in TauCl-treated mouse skin. (**A**) Skin thickness (double-headed arrow) and leukocyte infiltration (arrow head) were detected by H&E staining. Immunohistochemical analysis of 4-HNE-modified proteins (brown spots) and TUNEL-positive apoptotic cells in mouse epidermis. Magnification × 100. (**B**) The expression levels of 4-HNE-modified protein in UVB-irradiated mouse skin tissue with and without TauCl application were determined by Western blot analysis. Actin was used as an equal loading control. (**C**) Apoptosis was detected by measuring cleavage of caspase-3 by Western blot analysis. Data are analyzed by one-way ANOVA and expressed as means ± SD (*n* = 3 per group). *^,^ ** Significantly different between groups compared (* *p* < 0.05; ** *p* < 0.01).

Exposure to UVB irradiation leads to cutaneous inflammation and oxidative stress [6,7,8]. The images of H&E stained epidermal skin sections demonstrated attenuation of the skin hyperplasia and infiltration of inflammatory cells in TauCl-treated mice (Figure 1A). ROS-mediated oxidative stress causes lipid peroxidation and apoptotic DNA fragmentation [22,23]. The accumulation of 4-HNE, the end-product of lipid peroxidation, was abrogated by topical application of TauCl (Figure 1A,B). TauCl treatment also reduced the production of TUNEL-positive apoptotic cells (Figure 1A) and the cleavage of caspase-3, a hallmark of apoptotic cell death, in whole lysates (Figure 1C).

### 3.2. UVB-Induced Acute Skin Inflammation Was Ameliorated in TauCl-Treated Mice Skin

**Figure 2:**
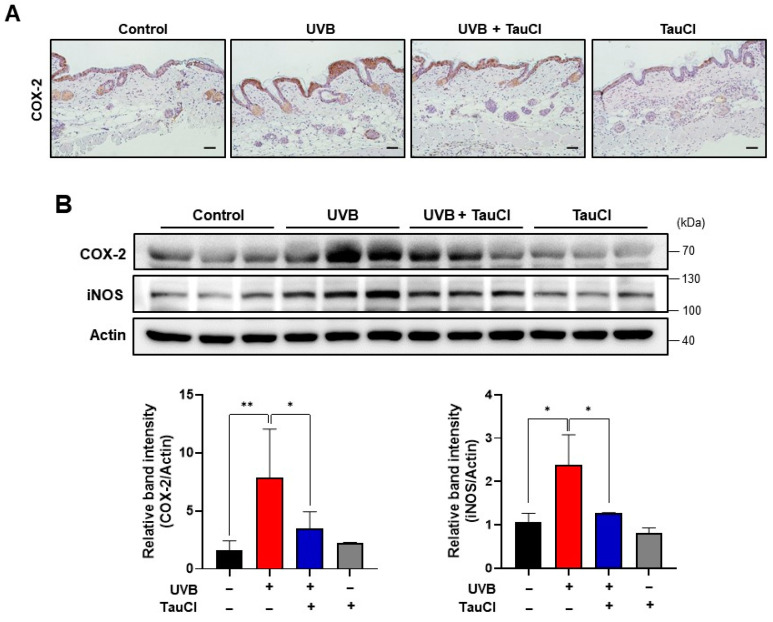
The expression of UVB-induced inflammatory enzymes was attenuated by topical application of TauCl. (**A**) Immunohistochemical analysis was performed for detecting COX-2 as described in Materials and Methods. (**B**) The expression levels of pro-inflammatory enzymes COX-2 and iNOS were measured by Western blot analysis of whole protein lysates. Actin was used as an equal loading control. Results are analyzed by one-way ANOVA and expressed as means ± SD (*n* = 3 per group. *^,^ ** Significantly different between groups compared (* *p* < 0.05; ** *p* < 0.01).

**Figure 3:**
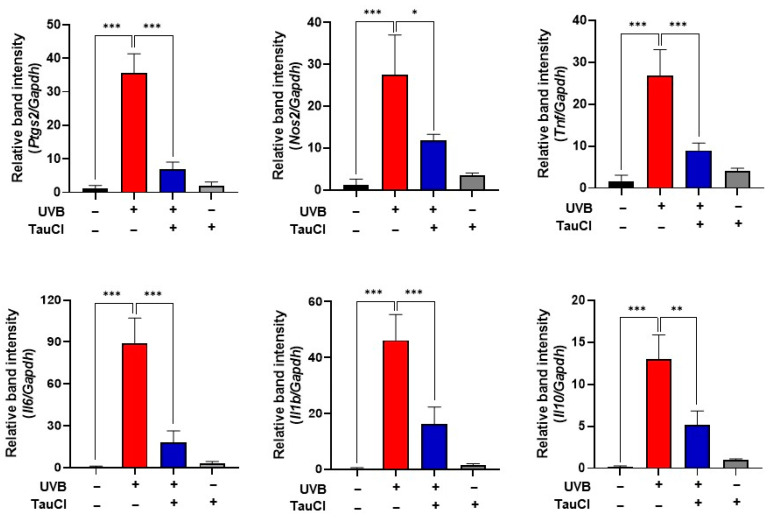
TauCl application inhibited UVB-induced expression of pro-inflammatory cytokines and enzymes in mouse skin. qPCR analysis of mRNA transcripts for pro-inflammatory cytokines (*Tnf, Il6, Il1b, Il10*) and enzymes (*Ptgs2, Nos2*). The treatment conditions and other experimental details are as described in Materials and Methods. Results are analyzed by one-way ANOVA and expressed as means ± SD (*n* = 3 per group). *^,^ **^,^ *** Significantly different between groups compared (* *p* < 0.05; ** *p* < 0.01; *** *p* < 0.001).

Aberrant overexpression of COX-2 and iNOS has been reported in murine models of dermatitis and photocarcinogenesis induced by UVB irradiation [24,25]. Immunohistochemical analysis was performed for detecting the expression level of COX-2 in mouse skin. As shown in Figure 2A, topical application of TauCl ameliorated UVB-induced expression of COX-2, and this was verified by Western blot analysis (Figure 2B). The UVB-induced expression of iNOS, another prototypic pro-inflammatory enzyme, was also attenuated by topically applied TauCl (Figure 2B). The analyses were performed with total protein lysates. Similarly, expression of genes encoding inflammation-associated cytokines (*Tnf, Il6, Il1b* and *Il10*), as well as *Ptgs2* and *Nos2* encoding COX-2 and iNOS, respectively, was diminished by TauCl treatment (Figure 3).

### 3.3. UVB-Induced Phosphorylation of STAT3 Was Blunted by Topical Application of TauCl

**Figure 4:**
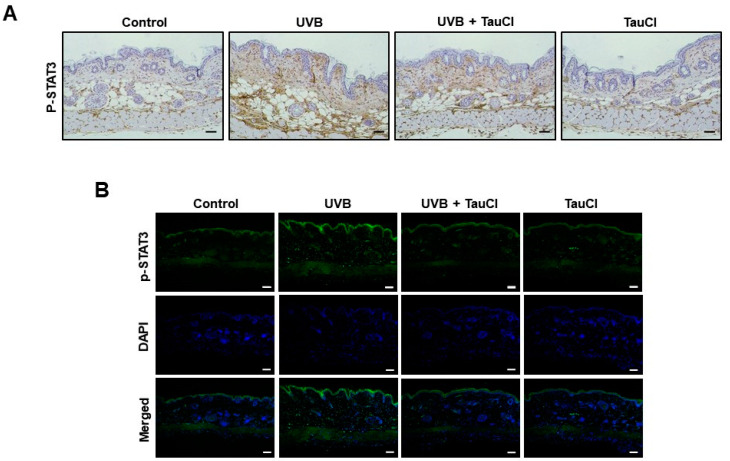
Topically applied TauCl attenuated UVB-induced phosphorylation of STAT3 in mouse skin. (**A**,**B**) Expression of P-STAT3 was measured by immunohistochemical analysis (**A**) and immunofluorescence staining (**B**). Scale bar, 200 μm.

**Figure 5:**
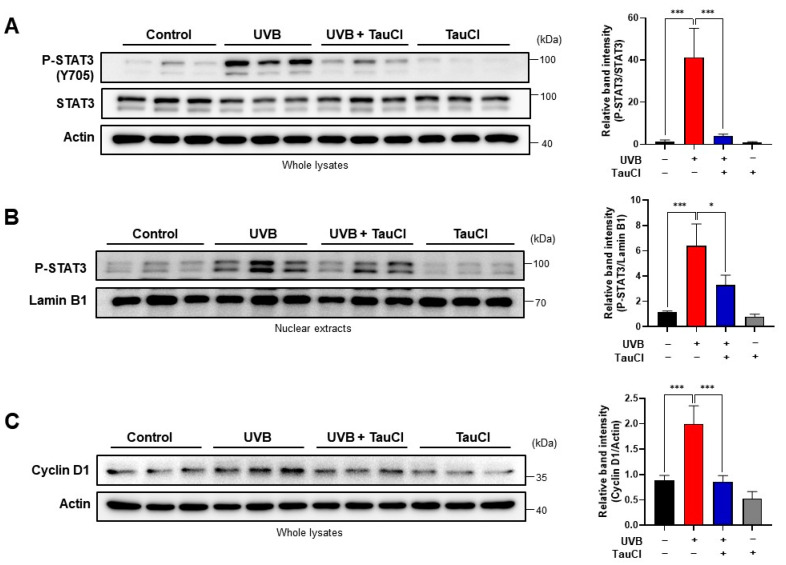
UVB-induced phosphorylation of STAT3 was diminished in TauCl-treated mouse skin. (**A**,**B**) The expression (**A**) and nuclear accumulation (**B**) of the phosphorylated STAT3 was measured by Western blot analysis. (**C**) The expression of Cyclin D1, a principal target protein of P-STAT3, was measured by Western blot analysis. Actin was used as an equal loading control. Data are expressed as means ± SD (*n* = 3 per group), analyzed by one-way ANOVA. *^,^ *** Significantly different between groups compared (* *p* < 0.05; *** *p* < 0.001).

STAT3 is a key transcription factor that regulates immunity and inflammation by promoting pro-inflammatory factors [26,27]. The phosphorylation of the tyrosine 705 residue of STAT3 facilitates its translocation into the nucleus, where it regulates the transcription of the target genes. The topical application of TauCl reduced the expression of phosphorylated STAT3 (P-STAT3) in mouse skin, as measured by immunohistochemical analysis (Figure 4A) and immunofluorescence staining (Figure 4B), which was confirmed by Western blot analysis (Figure 5A). TauCl also inhibited the nuclear translocation of P-STAT3 in UVB-irradiated mouse skin (Figure 5B). The expression of cyclin D1, a major target of P-STAT3, was also decreased in the same tendency (Figure 5C).

### 3.4. Topical Application of TauCl Upregulates Cytoprotective Gene Expression through Nrf2 Activation

**Figure 6:**
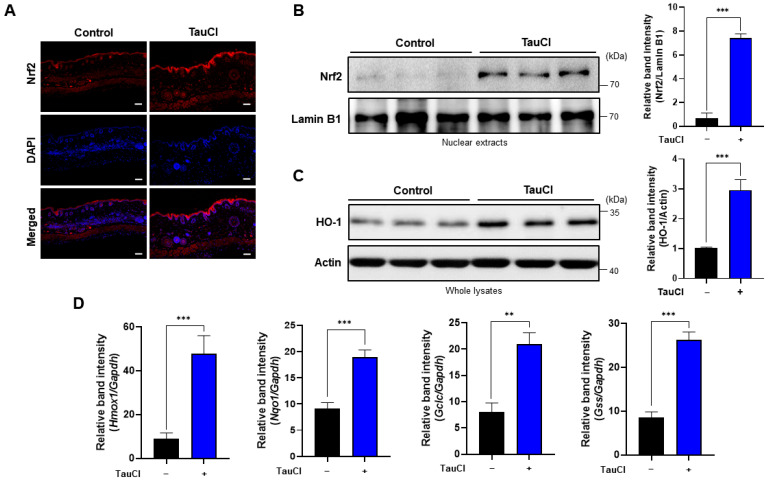
The Nrf2-mediated expression of antioxidant enzymes was elevated in TauCl-treated mice. (**A**,**B**) Nuclear localization of Nrf2 was assessed by immunofluorescence staining (**A**) and Western blot analysis (**B**). (**C**) The expression of HO-1 was determined by Western blot analysis. (**D**) Nrf2-mediated antioxidant gene expression was measured by qPCR. Results are expressed as means ± SD (*n* = 3 for each group), analyzed by Student’s *t*-test. **^,^ *** Significantly different between groups compared (** *p* < 0.01; *** *p* < 0.001). Scale bar, 200 µm.

Homeostatic imbalance is implicated in the pathogenesis of various disorders, such as autoimmune disease, inflammation, and tumorigenesis [28]. The master transcription factor Nrf2 plays a prominent role in the transcriptional regulation of stress-responsive genes, many of which encode antioxidant and anti-inflammatory proteins, including HO-1. The elevated expression of Nrf2 in the TauCl-treated mouse skin tissue was revealed by immunofluorescence staining (Figure 6A), and the localization of Nrf2 in the nucleus was confirmed by Western blot analysis (Figure 6B). There was a pronounced increase in the expression of HO-1 (Figure 6C) and its mRNA transcript, as well as transcription of other Nrf2 target antioxidant genes (Figure 6D).

## 4. Discussion

Taurine, also called 2-aminoethylsulfonic acid, is one of the amino sulfonic acids involved in various physiologic and pharmacologic processes [14,29,30]. It is found in organs through which a large amount of blood flow passes including the brain and heart, and it is also in high concentrations in some immune cells, especially neutrophils and monocytes [14]. From a microscopic point of view, taurine regulates the balance of electrolytes and minerals in cells, but from a more expanded macro point of view, it supports the immune system through antioxidant and anti-inflammatory functions. The health beneficial effects of taurine have been extensively investigated and well documented, and dietary supplements containing taurine are popular [30,31].

Though taurine provides diverse health benefits, there is a paucity of data in regard to its photoprotective effects. In one study, accumulation of taurine protected keratinocytes from UVB-induced apoptosis by regulating the osmosis through taurine transporter (TAUT) in human skin [32]. In cases of psoriasis patients, it was confirmed that the concentration of neutrophil taurine was lower as compared with normal subjects, suggesting the role for taurine in the body’s defense against skin inflammation [33]. Taurine reduced the generation of malondialdehyde, a marker for oxidative stress, and enhanced the collagen synthesis and the tensile strength, thereby accelerating wound healing [34].

Unlike chemically induced skin inflammation, the UVB-induced murine dermatitis model is useful in identifying the photoprotective substances and investigating their underlying molecular mechanisms [5,35,36]. Local skin inflammation caused by UVB irradiation is often associated with activation of NF-κB and STAT3 responsible for transcriptional regulation of diverse proinflammatory cytokines and enzymes, including COX-2 and iNOS [37,38,39]. ROS is inevitably produced by oxidative phosphorylation in mitochondria as well as metabolism of xenobiotics and some endogenous substances, particularly those undergoing redox cycling. Under physiological conditions, the generation of ROS is balanced by the cellular antioxidant defense system. However, the redox balance is disrupted by external factors, such as air pollution, ionizing radiation, microbial infection, chemicals, etc., which lead to DNA damage or cell death [40,41,42]. In the short term, ROS-mediated oxidative stress evokes oxidative damage to biomolecules, including membrane lipids. In the long term, it causes photoaging and photocarcinogenesis.

While it is unclear how taurine exerts a protective effect against oxidative damage to various tissues in the human body including the skin, its efficacy has been evaluated largely in animal studies. Under excessive oxidative stress or inflammatory conditions, the human body responds by stimulating immune cells and modulating expression/production of pro- and anti-inflammatory mediators [43]. ROS are formed in the process of inflammation-mediated tissue damage. Among these, hydrogen peroxide (H_2_O_2_) is converted to highly toxic HOCl through reaction with chloride ion, which is catalyzed by MPO [44,45]. Taurine reacts with HOCl to form TauCl, which is less toxic and has a strong antioxidant effect. TauCl not only ameliorates oxidative stress, but also suppresses the production of inflammatory mediators and stimulates the proliferation of activated immune cells [16,18,46,47].

Based on the research on antioxidant and anti-inflammatory actions exerted by taurine [13,14,48], we attempted to investigate the protective effect of TauCl on UVB-induced skin inflammation and underlying mechanisms. ROS production as a consequence of sustained UVB irradiation can cause oxidative stress and cell death [10]. In the present study, topical application of TauCl inhibited the expression of 4-HNE, the product of lipid peroxidation; cleavage of caspase-3, a marker for apoptosis; and activation of the pro-inflammatory transcription factor STAT3 and expression of its target protein, cyclin D1. It also suppressed the expression of COX-2, iNOS, and selected pro-inflammatory cytokines.

TauCl was found to regulate Nrf2-mediated antioxidant gene expression [18,49]. Our previous studies have demonstrated that TauCl potentiates the phagocytic and efferocytic capability of macrophages through Nrf2-mediated HO-1 upregulation [50,51]. In this study, the topical application of TauCl induced the accumulation of Nrf2 in the nucleus, thereby increasing the Nrf2-mediated anti-oxidant gene expression. The role of TauCl in phagocytic removal of apoptotic neutrophils, as well as dying epidermal cells in the context of resolution of UVB-irradiated mouse skin, merits further investigation.

## 5. Conclusions

**Figure 7:**
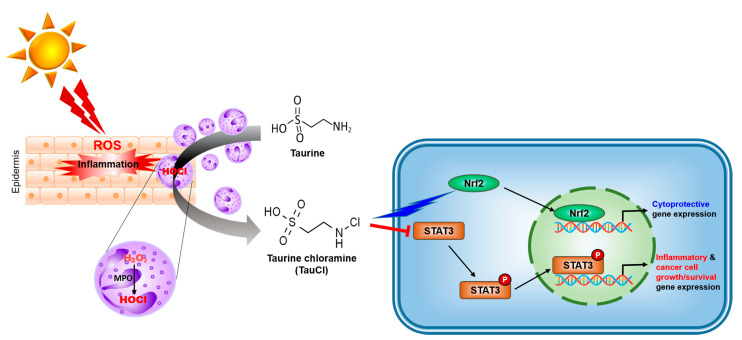
A proposed molecular mechanism underlying the protective effects of TauCl against UVB-induced dermatitis. Exposure to UVB causes inflammatory injury to the skin. Activated neutrophiles recruited to the inflamed site generate ROS, such as hydrogen peroxide, with concomitant release of myeloperoxidase (MPO). MPO catalyzes the reaction of hydrogen peroxide and chloride ion to produce hypochlorous acid (HOCl). HOCl is a strong oxidant and is neutralized by reacting with taurine, which produces TauCl. TauCl exerts antioxidant and anti-inflammatory effects by activating Nrf2 and blocking STAT3 signaling, leading to protection against UVB-induced skin damage.

TauCl reduces the expression of proinflammatory factors by fortifying antioxidant systems and inhibiting oxidative cell death in mouse skin. Thus, it protects against UVB-induced skin inflammation by promoting the expression of Nrf2-mediated antioxidant/anti-inflammatory enzymes, while suppressing pro-inflammatory gene expression (Figure 7). The amount of TauCl that is produced in response to inflammation is not sufficient enough to resolve inflammation effectively. In this context, direct application of exogenous TauCl onto skin can potentiate the cytoprotection against UVB-induced inflammation as well as oxidative tissue injury. The results of our present work suggest the possibility of TauCl as a lead compound in the development of effective therapeutics targeting dermatitis.

## Data Availability

Data presented in this study are included in the article.
